# Supplementary material for: Seasonal fluctuations of litter and soil Collembola and their drivers in rainforest and plantation systems
Source: PeerJ. 2024 Apr 1;12:e17125. doi: 10.7717/peerj.17125 (PMC10993886; doi:10.7717/peerj.17125)
Supplement: Supplemental Information 1 [file peerj-12-17125-s001.docx]

**Appendix**

**Appendix S1.** Seasonal variations in environmental factors in the litter layer of the three land-use systems studied (S1 - March, S2 - June, S3 - August, S4 – November) (data of four subplots per land-use system are given).

| System | Microbial carbon | Litter  pH | C/N  ratio | AMF marker | Fungal marker | Algae marker | Gram(-)  bacteria | Gram(+)  bacteria | Water content % | Fine root (<2mm) biomass (g) |
| --- | --- | --- | --- | --- | --- | --- | --- | --- | --- | --- |
| S1_Rainforest | 10933.8 | 4.52 | 23.6 | 6.35 | 49.80 | 0.13 | 30.17 | 45.38 | 81.8 | 0.79 |
| S1_Rainforest | 5741.0 | 4.02 | 28.3 | 4.78 | 73.04 | 0 | 34.57 | 38.61 | 144.21 | 2.73 |
| S1_Rainforest | 5809.2 | 3.53 | 24.4 | 6.93 | 66.91 | 0.21 | 44.50 | 59.19 | 154.14 | 7.3 |
| S1_Rainforest | 6176.0 | 3.61 | 31.7 | 5.54 | 77.31 | 0.03 | 38.76 | 50.24 | 97 | 2.65 |
| S1_Oil palm | 26171 | 5.22 | 27.0 | 8.49 | 59.56 | 0.33 | 52.11 | 84.09 | 59.96 | 0 |
| S1_Oil palm | 8614.2 | 5.25 | 28.9 | 6.12 | 51.01 | 0.01 | 34.72 | 53.64 | 13.87 | 2.59 |
| S1_Oil palm | 5146.8 | 4.25 | 25.7 | 2.98 | 89.46 | 0.01 | 21.07 | 34.94 | 26.39 | 1.38 |
| S1_Oil palm | 8951.9 | 5.93 | 25.2 | 6.71 | 30.12 | 0.79 | 60.19 | 102.56 | 7.95 | 1.13 |
| S1_Rubber | 16437.1 | 5.08 | 28.6 | 6.14 | 105.65 | 0.12 | 36.73 | 58.98 | 37.52 | 0 |
| S1_Rubber | 22668.1 | 4.98 | 23.4 | 8.88 | 25.80 | 0.07 | 35.52 | 56.48 | 33.31 | 0.34 |
| S1_Rubber | 13800.6 | 4.97 | 21.6 | 9.03 | 67.93 | 0.01 | 33.91 | 46.77 | 44.93 | 0.43 |
| S1_Rubber | 18053.6 | 4.78 | 28.5 | 6.72 | 89.53 | 0.01 | 42.57 | 55.80 | 54.83 | 1.85 |
| S2_Rainforest | 6931.7 | 3.96 | 33.2 | 4.44 | 60.62 | 0 | 45.51 | 37.96 | 17.91 | 0.35 |
| S2_Rainforest |  | 3.92 | 25.7 | 4.79 | 37.67 | 0 | 44.61 | 43.61 | 151.55 | 0.83 |
| S2_Rainforest | 14613.3 | 3.96 | 25.6 | 6.65 | 51.52 | 0 | 48.76 | 53.86 | 65.57 | 1.7 |
| S2_Oil palm | 6159.5 | 5.3 | 26.2 | 4.57 | 52.07 | 0 | 30.80 | 36.32 | 55.58 | 0.04 |
| S2_Oil palm | 4138.6 | 4.84 | 32.7 | 4.57 | 41.91 | 0 | 27.55 | 34.23 | 22.4 | 0.75 |
| S2_Oil palm | 5595.9 | 4.62 | 24.7 | 3.07 | 123.72 | 0 | 67.28 | 35.63 | 39.15 | 0.42 |
| S2_Oil palm | 5600.2 | 6.18 | 36.8 | 6.96 | 93.47 | 0 | 90.21 | 72.89 | 22.92 | 0.49 |
| S2_Rubber | 8598.9 | 5.27 | 28.4 | 6.35 | 101.65 | 0 | 65.54 | 45.84 | 20.99 | 0.18 |
| S2_Rubber | 13656.4 | 5.17 | 27.2 | 4.25 | 57.39 | 0 | 46.52 | 46.72 | 35.27 | 1.17 |
| S2_Rubber | 7568.0 | 4.97 | 32.4 | 2.74 | 101.19 | 0 | 39.99 | 27.39 | 38.09 | 0.14 |
| S2_Rubber | 6007.0 | 4.5 | 30.6 | 2.88 | 109.04 | 0 | 44.57 | 33.10 | 41.28 | 0.38 |
| S3_Rainforest | 6742.1 | 4.47 | 27.6 | 2.13 | 32.46 | 0 | 30.29 | 21.14 | 64.98 | 0.36 |
| S3_Rainforest | 5578.3 | 4.11 | 30.3 | 2.25 | 22.06 | 0 | 24.10 | 27.51 | 68.27 | 1.25 |
| S3_Rainforest | 5644.5 | 4.26 | 27.2 | 3.50 | 30.64 | 0 | 32.66 | 30.89 | 59.22 | 2.65 |
| S3_Oil palm | 6264.3 | 3.63 | 31.8 | 3.18 | 23.74 | 0 | 29.57 | 31.95 | 72.82 | 1.74 |
| S3_Oil palm | 6806.5 | 5.29 | 35.5 | 2.83 | 88.67 | 0 | 18.65 | 26.07 | 55.9 | 0.01 |
| S3_Oil palm | 9068.9 | 6.52 | 38.1 | 6.01 | 62.39 | 0 | 55.53 | 61.69 | 25.33 | 0 |
| S3_Oil palm | 3377.8 | 4.35 | 24.5 | 5.27 | 123.79 | 0 | 21.55 | 29.32 | 25.65 | 0.42 |
| S3_Rubber | 4896.3 | 4.76 | 41.6 | 3.14 | 75.89 | 0 | 17.58 | 25.29 | 74.69 | 0.04 |
| S3_Rubber | 8320.4 | 5.65 | 35.1 | 2.27 | 45.16 | 0 | 29.48 | 17.50 | 88.61 | 0.19 |
| S3_Rubber | 18763.4 | 5.8 | 28.9 | 3.87 | 0 | 0 | 30.97 | 20.08 | 77.42 | 0.07 |
| S3_Rubber | 7019.1 | 5.6 | 35.1 | 6.25 | 0 | 1.02 | 79.27 | 64.59 | 88.36 | 0 |
| S4_Rainforest | 4200.8 | 4.42 | 31.4 | 4.13 | 104.68 | 0 | 112.19 | 63.34 | 50.19 | 3 |
| S4_Rainforest | 7296.1 | 4.61 | 26.1 | 2.61 | 37.42 | 0 | 48.25 | 38.45 | 64.95 | 0.1 |
| S4_Rainforest | 5176.3 | 4.21 | 30.1 | 1.58 | 40.10 | 0 | 27.18 | 17.88 | 200.7 | 0 |
| S4_Rainforest | 11062.7 | 4.26 | 31.1 | 2.22 | 20.85 | 0 | 58.53 | 24.29 | 122.02 | 1.47 |
| S4_Oil palm | 14587.5 | 3.99 | 31.9 | 1.97 | 24.42 | 0 | 86.87 | 13.23 | 68.49 | 0.67 |
| S4_Oil palm | 25157.7 | 5.63 | 24.3 | 3.30 | 11.46 | 0 | 37.61 | 75.01 | 66.25 | 0.13 |
| S4_Oil palm | 16624.2 | 5.34 | 28.3 | 3.07 | 26.56 | 0 | 33.68 | 49.29 | 71.81 | 0.62 |
| S4_Oil palm | 3947.1 | 4.94 | 21.4 | 2.61 | 44.25 | 0 | 49.94 | 52.19 | 124.54 | 0.41 |
| S4_Rubber | 10480.4 | 5.74 | 26.5 | 4.94 | 98.94 | 0 | 87.86 | 131.69 | 91.27 | 0.3 |
| S4_Rubber | 13253.1 | 5.78 | 22.8 | 7.83 | 114.70 | 0 | 71.61 | 95.45 | 109.29 | 0.14 |
| S4_Rubber | 7141.5 | 5.51 | 22.8 | 11.28 | 48.27 | 0 | 73.41 | 132.21 | 62.15 | 1.77 |
| S4_Rubber | 15062.9 | 5.03 | 23.6 | 9.94 | 74.91 | 0 | 132.92 | 143.57 | 115.26 | 0.86 |

**Appendix S2.** Seasonal variations in environmental factors in the soil layer of the three land-use systems studied (S1 - March, S2 - June, S3 - August, S4 – November) (data of four subplots per land-use system are given).

| System | Microbial  carbon | Soil  pH | C/N ratio | AMF marker | Fungal marker | Algae marker | Gram(-) bacteria | Gram(+)  bacteria | Water content % | Root biomass (g) |
| --- | --- | --- | --- | --- | --- | --- | --- | --- | --- | --- |
| S1_Rainforest | 570.5 | 4 | 14.8 | 1.57 | 1.82 | 0 | 8.15 | 20.53 | 242.33 | 4.59 |
| S1_Rainforest | 477.1 | 3.92 | 13.8 | 1.21 | 0.90 | 0 | 0.91 | 21.21 | 269.24 | 3.26 |
| S1_Rainforest | 475.7 | 3.63 | 14.7 | 2.10 | 1.35 | 0 | 2.21 | 24.06 | 267.36 | 4.44 |
| S1_Rainforest | 618.3 | 3.63 | 14.9 | 2.20 | 1.74 | 0 | 11.59 | 24.96 | 242.9 | 3.97 |
| S1_Oil palm | 836.0 | 5.44 | 13.3 | 4.23 | 2.41 | 0.03 | 14.07 | 32.32 | 248.07 | 10.32 |
| S1_Oil palm | 213.6 | 4.35 | 12.7 | 1.77 | 1.14 | 0.01 | 2.81 | 17.02 | 263.24 | 6.17 |
| S1_Oil palm | 266.0 | 4.37 | 13.3 | 0.40 | 0.18 | 0 | 4.12 | 12.12 | 241.33 | 1.21 |
| S1_Oil palm | 866.5 | 4.85 | 16.8 | 2.11 | 1.00 | 0.01 | 8.39 | 23.88 | 246.97 | 8.77 |
| S1_Rubber | 309.6 | 4.14 | 11.8 | 1.92 | 1.10 | 0 | 3.99 | 17.94 | 243.56 | 1.75 |
| S1_Rubber | 515.7 | 4.16 | 11.5 | 2.10 | 0 | 0 | 4.77 | 21.32 | 247.47 | 1.51 |
| S1_Rubber | 317.4 | 4.24 | 13 | 1.45 | 0.31 | 0 | 1.88 | 17.39 | 235.54 | 3.17 |
| S1_Rubber | 623.5 | 4.12 | 12.9 | 2.91 | 0 | 0 | 6.31 | 31.70 | 241.22 | 3.75 |
| S2_Rainforest | 646.9 | 3.9 | 14.1 | 2.42 | 1.62 | 0 | 11.79 | 24.37 | 245.07 | 1.34 |
| S2_Rainforest | 459.6 | 3.68 | 15.6 | 2.62 | 1.14 | 0 | 19.07 | 40.78 | 247.24 | 4.07 |
| S2_Rainforest |  | 3.39 | 17.1 | 3.37 | 1.76 | 0 | 22.84 | 36.37 | 233.16 | 4.8 |
| S2_Rainforest | 602.7 | 3.49 | 15.4 | 3.44 | 2.78 | 0 | 20.69 | 32.57 | 267.49 | 5.29 |
| S2_Oil palm | 502.5 | 4.74 | 12.9 | 2.42 | 1.05 | 0 | 8.19 | 22.47 | 258.77 | 5.63 |
| S2_Oil palm | 246.4 | 4.3 | 11.7 | 1.12 | 0.56 | 0 | 2.71 | 11.57 | 267.21 | 5.1 |
| S2_Oil palm | 181.4 | 4.42 | 12.3 | 2.48 | 0.82 | 0 | 7.25 | 22.75 | 255.21 | 1.08 |
| S2_Oil palm | 426.9 | 4.44 | 14.5 | 1.30 | 0 | 0 | 5.69 | 13.84 | 233.61 | 5.39 |
| S2_Rubber | 348.9 | 4.24 | 11.8 | 2.13 | 1.03 | 0 | 6.18 | 17.51 | 273.88 | 1.85 |
| S2_Rubber | 626.6 | 4.12 | 11.4 | 0 | 0 | 0 | 2.90 | 12.61 | 267.17 | 2.51 |
| S2_Rubber | 246.4 | 4.15 | 13 | 1.58 | 0 | 0 | 4.29 | 15.15 | 250.51 | 1.07 |
| S2_Rubber | 388.1 | 4.05 | 12.6 | 2.82 | 0.63 | 0 | 6.33 | 26.71 | 255.5 | 2.36 |
| S3_Rainforest | 329.9 | 3.76 | 14.9 | 2.60 | 1.15 | 0 | 13.58 | 29.07 | 267.17 | 1.71 |
| S3_Rainforest | 436.1 | 3.72 | 14 | 2.47 | 2.71 | 0 | 12.40 | 26.74 | 272.2 | 4.99 |
| S3_Rainforest | 353.2 | 3.61 | 15.9 | 3.18 | 2.13 | 0 | 18.01 | 33.52 | 247.97 | 5.39 |
| S3_Oil palm | 711.7 | 4.4 | 14.8 | 2.89 | 0.97 | 0 | 10.90 | 29.55 | 251.17 | 4.45 |
| S3_Oil palm | 266.9 | 4.48 | 15.7 | 3.07 | 1.67 | 0 | 10.21 | 25.69 | 234.43 | 8.11 |
| S3_Oil palm | 156.0 | 4.39 | 13.2 | 0 | 0 | 0 | 2.24 | 10.17 | 249.66 | 0.95 |
| S3_Oil palm | 220.5 | 4.15 | 15 | 1.55 | 0 | 0 | 4.84 | 17.62 | 251.95 | 4.82 |
| S3_Rubber | 518.0 | 4.22 | 11.1 | 2.56 | 0.92 | 0 | 12.57 | 24.17 | 243.47 | 2.52 |
| S3_Rubber | 459.0 | 4.13 | 12.1 | 3.89 | 1.25 | 0 | 16.42 | 36.59 | 262.21 | 4.7 |
| S3_Rubber | 235.8 | 4.19 | 12.2 | 1.63 | 0 | 0 | 6.06 | 16.72 | 242.5 | 1.28 |
| S3_Rubber | 561.9 | 4.13 | 13.8 | 2.55 | 0.75 | 0 | 9.26 | 28.96 | 233.25 | 1.08 |
| S4_Rainforest | 258.3 | 4.05 | 13.0 | 0.76 | 0 | 0 | 11.40 | 17.33 | 258.02 | 3.24 |
| S4_Rainforest | 451.7 | 3.78 | 15.9 | 0.50 | 0 | 0 | 8.24 | 8.77 | 225.68 | 2.39 |
| S4_Rainforest | 250.7 | 4.14 | 15.1 | 2.17 | 0.84 | 0 | 13.06 | 26.01 | 239.31 | 6.63 |
| S4_Rainforest | 351.2 | 3.7 | 15.5 | 1.87 | 0.70 | 0 | 13.68 | 24.21 | 257.94 | 4.52 |
| S4_Oil palm | 345.5 | 4.2 | 14.2 | 0.42 | 0.57 | 0 | 2.83 | 2.53 | 250.62 | 5.27 |
| S4_Oil palm | 179.2 | 4.63 | 13.6 | 0.80 | 0 | 0 | 2.06 | 5.51 | 265.88 | 5.28 |
| S4_Oil palm | 98.8 | 4.38 | 12.6 | 0 | 0 | 0 | 0.52 | 0.16 | 267.07 | 1.72 |
| S4_Oil palm | 308.3 | 4.53 | 16.3 | 0.63 | 0.51 | 0 | 8.27 | 12.25 | 255.81 | 4.72 |
| S4_Rubber | 450.1 | 4.2 | 10.9 | 0.43 | 0.86 | 0 | 3.21 | 3.94 | 259.38 | 1.15 |
| S4_Rubber | 267.1 | 4.16 | 11.1 | 0.98 | 0 | 0 | 4.73 | 6.58 | 236.05 | 1.28 |
| S4_Rubber | 227.9 | 4.28 | 11.6 | 0.45 | 0 | 0 | 4.04 | 2.85 | 243.87 | 2.23 |
| S4_Rubber | 455.2 | 4.05 | 13.7 | 0.89 | 0 | 0 | 3.24 | 9.32 | 249.35 | 1.4 |

**Appendix S3.** Species matrix of Collembola in the litter layer in rainforest, rubber and oil palm plantations at four sampling dates (S1 - March, S2 - June, S3 - August, S4 - November) (data of four subplots per land-use system are given).

| System | Acr | Asc | Folc | Folp | Foln | Hom | Isolu | Isomi | Isosy | Lep | Meg | Par | Pro | Psed | Pseu | Pte | Ram | Sal | Sm.g | Sph1 | Sph2 | Sphy | Tha | |
| --- | --- | --- | --- | --- | --- | --- | --- | --- | --- | --- | --- | --- | --- | --- | --- | --- | --- | --- | --- | --- | --- | --- | --- | --- |
| S1_Rainforest | 0 | 0 | 7 | 72 | 8 | 0 | 0 | 24 | 7 | 0 | 31 | 0 | 0 | 0 | 40 | 0 | 1 | 0 | 15 | 30 | 0 | 0 | 0 |  |
| S1_Rainforest | 0 | 32 | 0 | 2 | 0 | 0 | 0 | 15 | 10 | 0 | 13 | 0 | 0 | 0 | 95 | 0 | 2 | 0 | 25 | 0 | 0 | 0 | 0 |  |
| S1_Rainforest | 0 | 29 | 0 | 1 | 14 | 0 | 19 | 13 | 0 | 0 | 39 | 3 | 1 | 0 | 178 | 0 | 1 | 0 | 10 | 10 | 11 | 0 | 16 |  |
| S1_Rainforest | 1 | 26 | 2 | 7 | 13 | 0 | 9 | 0 | 0 | 0 | 0 | 7 | 0 | 0 | 6 | 0 | 4 | 0 | 0 | 4 | 0 | 0 | 5 |  |
| S1_Oil palm | 1 | 0 | 19 | 3 | 0 | 0 | 6 | 0 | 0 | 1 | 0 | 0 | 0 | 1 | 4 | 3 | 0 | 0 | 0 | 0 | 0 | 0 | 0 |  |
| S1_Oil palm | 0 | 0 | 11 | 0 | 0 | 2 | 0 | 0 | 0 | 0 | 0 | 0 | 0 | 0 | 0 | 0 | 0 | 0 | 0 | 0 | 0 | 0 | 0 |  |
| S1_Oil palm | 0 | 1 | 4 | 3 | 0 | 0 | 4 | 0 | 0 | 0 | 0 | 0 | 0 | 0 | 0 | 1 | 0 | 1 | 0 | 0 | 0 | 0 | 0 |  |
| S1_Oil palm | 0 | 0 | 5 | 0 | 0 | 1 | 1 | 0 | 0 | 0 | 0 | 0 | 0 | 0 | 0 | 1 | 0 | 0 | 0 | 0 | 0 | 0 | 0 |  |
| S1_Rubber | 0 | 0 | 1 | 1 | 0 | 3 | 0 | 0 | 0 | 0 | 0 | 0 | 0 | 0 | 0 | 0 | 0 | 1 | 0 | 0 | 0 | 0 | 0 |  |
| S1_Rubber | 0 | 0 | 10 | 1 | 0 | 0 | 7 | 0 | 0 | 3 | 0 | 0 | 0 | 0 | 1 | 0 | 0 | 0 | 3 | 0 | 0 | 5 | 0 |  |
| S1_Rubber | 0 | 0 | 19 | 3 | 0 | 3 | 0 | 0 | 0 | 0 | 0 | 0 | 0 | 0 | 0 | 0 | 0 | 0 | 0 | 0 | 0 | 0 | 0 |  |
| S1_Rubber | 0 | 3 | 4 | 3 | 0 | 0 | 1 | 0 | 0 | 0 | 0 | 0 | 0 | 0 | 1 | 0 | 0 | 0 | 0 | 2 | 0 | 0 | 0 |  |
| S2_Rainforest | 0 | 1 | 0 | 0 | 0 | 0 | 0 | 0 | 0 | 0 | 0 | 0 | 0 | 0 | 0 | 0 | 0 | 0 | 0 | 0 | 0 | 0 | 0 |  |
| S2_Rainforest | 1 | 92 | 3 | 6 | 1 | 0 | 9 | 0 | 0 | 0 | 59 | 0 | 0 | 14 | 114 | 0 | 53 | 0 | 9 | 5 | 4 | 13 | 1 |  |
| S2_Rainforest | 0 | 0 | 15 | 5 | 5 | 0 | 4 | 0 | 5 | 1 | 19 | 0 | 0 | 0 | 95 | 0 | 0 | 1 | 5 | 9 | 0 | 3 | 6 |  |
| S2_Oil palm | 0 | 0 | 41 | 10 | 0 | 0 | 5 | 4 | 0 | 0 | 0 | 0 | 0 | 0 | 11 | 4 | 0 | 0 | 2 | 2 | 0 | 0 | 3 |  |
| S2_Oil palm | 0 | 0 | 61 | 1 | 0 | 0 | 0 | 0 | 0 | 0 | 0 | 0 | 0 | 0 | 0 | 1 | 0 | 0 | 2 | 0 | 0 | 0 | 0 |  |
| S2_Oil palm | 21 | 0 | 49 | 5 | 0 | 1 | 0 | 0 | 0 | 0 | 0 | 0 | 0 | 0 | 6 | 10 | 0 | 0 | 12 | 0 | 0 | 0 | 0 |  |
| S2_Oil palm | 0 | 0 | 9 | 0 | 0 | 0 | 2 | 0 | 0 | 0 | 0 | 0 | 0 | 0 | 3 | 0 | 0 | 0 | 0 | 0 | 0 | 0 | 1 |  |
| S2_Rubber | 0 | 5 | 0 | 0 | 0 | 0 | 3 | 0 | 0 | 0 | 4 | 0 | 0 | 0 | 4 | 0 | 0 | 0 | 2 | 0 | 0 | 0 | 1 |  |
| S2_Rubber | 5 | 7 | 6 | 1 | 0 | 0 | 2 | 0 | 0 | 0 | 0 | 0 | 0 | 0 | 2 | 2 | 0 | 0 | 9 | 1 | 6 | 1 | 0 |  |
| S2_Rubber | 0 | 7 | 14 | 0 | 0 | 1 | 0 | 0 | 0 | 0 | 0 | 0 | 0 | 0 | 0 | 1 | 0 | 0 | 5 | 2 | 0 | 0 | 0 |  |
| S2_Rubber | 0 | 45 | 19 | 3 | 0 | 0 | 11 | 21 | 0 | 0 | 24 | 1 | 3 | 0 | 19 | 1 | 0 | 0 | 10 | 7 | 0 | 1 | 0 |  |
| S3_Rainforest | 1 | 7 | 39 | 73 | 0 | 0 | 0 | 0 | 0 | 0 | 3 | 2 | 0 | 12 | 45 | 0 | 4 | 0 | 0 | 216 | 0 | 31 | 0 |  |
| S3_Rainforest | 0 | 24 | 0 | 22 | 0 | 0 | 32 | 0 | 0 | 0 | 17 | 0 | 0 | 0 | 33 | 0 | 0 | 2 | 0 | 35 | 0 | 0 | 0 |  |
| S3_Rainforest | 0 | 39 | 1 | 20 | 0 | 0 | 20 | 0 | 0 | 0 | 1 | 0 | 0 | 6 | 20 | 0 | 0 | 0 | 0 | 10 | 10 | 6 | 2 |  |
| S3_Oil palm | 1 | 14 | 0 | 0 | 0 | 0 | 0 | 0 | 0 | 0 | 2 | 3 | 0 | 0 | 6 | 0 | 0 | 0 | 0 | 10 | 13 | 0 | 0 |  |
| S3_Oil palm | 0 | 0 | 0 | 0 | 0 | 0 | 0 | 0 | 0 | 0 | 0 | 0 | 0 | 0 | 0 | 0 | 0 | 0 | 0 | 0 | 0 | 1 | 0 |  |
| S3_Oil palm | 0 | 0 | 0 | 0 | 0 | 0 | 0 | 0 | 0 | 0 | 0 | 0 | 0 | 0 | 0 | 0 | 0 | 0 | 0 | 0 | 0 | 0 | 2 |  |
| S3_Oil palm | 0 | 0 | 1 | 0 | 0 | 0 | 0 | 0 | 0 | 0 | 0 | 0 | 0 | 0 | 0 | 0 | 0 | 0 | 3 | 0 | 0 | 0 | 0 |  |
| S3_Rubber | 0 | 0 | 1 | 0 | 0 | 0 | 0 | 0 | 0 | 1 | 0 | 0 | 0 | 0 | 0 | 0 | 0 | 0 | 4 | 0 | 0 | 0 | 0 |  |
| S3_Rubber | 0 | 31 | 0 | 0 | 0 | 0 | 17 | 0 | 10 | 0 | 6 | 26 | 0 | 5 | 14 | 0 | 0 | 0 | 0 | 0 | 4 | 0 | 0 |  |
| S3_Rubber | 0 | 4 | 28 | 19 | 0 | 0 | 7 | 0 | 0 | 8 | 0 | 6 | 0 | 0 | 0 | 3 | 0 | 0 | 0 | 9 | 19 | 28 | 1 |  |
| S3_Rubber | 0 | 6 | 4 | 1 | 2 | 0 | 8 | 3 | 0 | 0 | 1 | 0 | 1 | 0 | 66 | 0 | 8 | 0 | 0 | 0 | 0 | 0 | 0 |  |
| S4_Rainforest | 0 | 7 | 0 | 0 | 0 | 21 | 0 | 0 | 0 | 0 | 0 | 0 | 0 | 0 | 0 | 0 | 0 | 4 | 0 | 1 | 0 | 0 | 0 |  |
| S4_Rainforest | 0 | 2 | 3 | 6 | 0 | 0 | 0 | 15 | 7 | 2 | 21 | 2 | 1 | 0 | 61 | 0 | 11 | 0 | 39 | 0 | 43 | 0 | 1 |  |
| S4_Rainforest | 0 | 69 | 3 | 6 | 2 | 0 | 0 | 0 | 18 | 0 | 29 | 11 | 0 | 0 | 63 | 0 | 5 | 1 | 8 | 0 | 17 | 0 | 0 |  |
| S4_Rainforest | 0 | 28 | 4 | 0 | 0 | 0 | 0 | 17 | 0 | 16 | 30 | 5 | 0 | 4 | 30 | 0 | 1 | 0 | 0 | 68 | 0 | 0 | 0 |  |
| S4_Oil palm | 0 | 43 | 4 | 2 | 0 | 0 | 0 | 0 | 11 | 0 | 6 | 2 | 0 | 1 | 31 | 0 | 7 | 0 | 0 | 53 | 0 | 8 | 0 |  |
| S4_Oil palm | 0 | 0 | 75 | 6 | 0 | 0 | 2 | 0 | 0 | 0 | 3 | 0 | 0 | 0 | 7 | 0 | 0 | 0 | 0 | 1 | 0 | 0 | 0 |  |
| S4_Oil palm | 0 | 1 | 50 | 2 | 0 | 0 | 1 | 0 | 0 | 0 | 2 | 0 | 0 | 0 | 9 | 0 | 5 | 4 | 0 | 0 | 0 | 0 | 0 |  |
| S4_Oil palm | 0 | 0 | 10 | 0 | 0 | 0 | 10 | 0 | 0 | 2 | 0 | 0 | 0 | 0 | 1 | 0 | 0 | 0 | 0 | 0 | 0 | 0 | 0 |  |
| S4_Rubber | 0 | 1 | 45 | 10 | 0 | 0 | 0 | 0 | 0 | 2 | 0 | 0 | 0 | 1 | 23 | 2 | 0 | 0 | 0 | 1 | 0 | 2 | 0 |  |
| S4_Rubber | 0 | 1 | 14 | 0 | 0 | 0 | 0 | 76 | 44 | 6 | 54 | 0 | 0 | 3 | 3 | 0 | 0 | 0 | 0 | 3 | 0 | 0 | 2 |  |
| S4_Rubber | 2 | 1 | 16 | 47 | 0 | 1 | 0 | 0 | 8 | 3 | 1 | 0 | 0 | 0 | 0 | 2 | 0 | 0 | 0 | 0 | 1 | 0 | 1 |  |
| S4_Rubber | 0 | 14 | 4 | 0 | 0 | 1 | 0 | 56 | 0 | 0 | 40 | 0 | 3 | 0 | 31 | 0 | 5 | 0 | 0 | 0 | 0 | 0 | 23 |  |

*Acr=Acrocyrtus sp.1, Asco=Ascocyrtus cinctus, , Folc=Folsomides centralis, Folsp=Folsomides parvulus, Foln=Folsomina onychiurina, Hom=Homidia cingula, Isolu=Isotomiella cf. alulu, Isomi=Isotomiella cf. minor, Isosys= Isotomiella cf. symetrimucronata, Lep=Lepidocyrtus sp.1, Meg=Megalothorax cf.minimus, Par=Pararrhopalites sp.1, Pro=Pronura sp.1, Psed= Pseudachorutes sp.1, Pseu=Pseudosinella sp.1, Pte=Ptenothrix sp.1, Ram=Rambutsinella sp.1, Sal=Salina sp.1, Sm.g=Sminthurides sp.1, Spha= Sphaeridia sp.1, Spha=Sphaeridia sp.2, Sphy= Sphyroteca sp.1, Xen= Xenylla sp.1*

**Appendix S4.** Species matrix of Collembola in the soil layer in rainforest, rubber and oil palm plantations at four sampling dates (S1 - March, S2 - June, S3 - August, S4 - November) (data of four subplots per land-use system are given).

| System | Acr | All | Asc | Folc | Folp | Foln | Hom | Isolu | Isodes | Meg | Pseu | Ram | Sphy | Xen |
| --- | --- | --- | --- | --- | --- | --- | --- | --- | --- | --- | --- | --- | --- | --- |
| S1_Rainforest | 0 | 6 | 0 | 0 | 1 | 0 | 0 | 3 | 0 | 3 | 12 | 0 | 0 | 1 |
| S1_Rainforest | 0 | 15 | 3 | 0 | 0 | 0 | 0 | 4 | 0 | 0 | 7 | 0 | 0 | 0 |
| S1_Rainforest | 0 | 10 | 7 | 5 | 0 | 10 | 0 | 0 | 0 | 4 | 69 | 0 | 0 | 3 |
| S1_Rainforest | 0 | 0 | 2 | 0 | 0 | 50 | 0 | 0 | 0 | 0 | 27 | 6 | 0 | 0 |
| S1_Oil palm | 5 | 12 | 0 | 1 | 0 | 0 | 0 | 0 | 1 | 0 | 14 | 0 | 0 | 0 |
| S1_Oil palm | 3 | 0 | 0 | 7 | 0 | 0 | 2 | 0 | 0 | 0 | 11 | 0 | 0 | 1 |
| S1_Oil palm | 1 | 0 | 0 | 3 | 1 | 0 | 0 | 3 | 17 | 0 | 2 | 0 | 0 | 0 |
| S1_Oil palm | 0 | 7 | 0 | 18 | 1 | 4 | 1 | 0 | 0 | 6 | 51 | 1 | 0 | 0 |
| S1_Rubber | 0 | 20 | 14 | 0 | 0 | 0 | 0 | 0 | 0 | 0 | 33 | 0 | 0 | 0 |
| S1_Rubber | 0 | 8 | 49 | 5 | 1 | 0 | 0 | 0 | 0 | 0 | 48 | 0 | 1 | 2 |
| S1_Rubber | 0 | 3 | 1 | 12 | 3 | 2 | 1 | 0 | 0 | 0 | 7 | 0 | 0 | 0 |
| S1_Rubber | 0 | 0 | 6 | 8 | 0 | 0 | 0 | 8 | 0 | 9 | 29 | 3 | 0 | 0 |
| S2_Rainforest | 1 | 1 | 0 | 5 | 0 | 0 | 0 | 4 | 0 | 4 | 70 | 5 | 1 | 0 |
| S2_Rainforest | 0 | 0 | 3 | 0 | 1 | 3 | 0 | 3 | 0 | 13 | 78 | 15 | 1 | 0 |
| S2_Rainforest | 0 | 0 | 7 | 0 | 1 | 9 | 0 | 6 | 0 | 0 | 50 | 0 | 0 | 1 |
| S2_Rainforest | 0 | 0 | 2 | 0 | 0 | 1 | 0 | 0 | 0 | 0 | 17 | 3 | 0 | 1 |
| S2_Oil palm | 10 | 6 | 0 | 1 | 3 | 0 | 0 | 2 | 2 | 3 | 24 | 0 | 0 | 0 |
| S2_Oil palm | 0 | 3 | 0 | 6 | 0 | 0 | 0 | 0 | 0 | 0 | 22 | 0 | 0 | 0 |
| S2_Oil palm | 1 | 0 | 4 | 3 | 0 | 0 | 1 | 0 | 0 | 0 | 41 | 0 | 0 | 0 |
| S2_Oil palm | 0 | 3 | 0 | 4 | 0 | 0 | 0 | 3 | 0 | 0 | 24 | 0 | 0 | 0 |
| S2_Rubber | 0 | 5 | 30 | 0 | 0 | 0 | 0 | 0 | 0 | 19 | 32 | 0 | 2 | 2 |
| S2_Rubber | 0 | 0 | 28 | 5 | 1 | 0 | 0 | 2 | 0 | 5 | 10 | 0 | 0 | 0 |
| S2_Rubber | 2 | 3 | 4 | 4 | 0 | 0 | 0 | 0 | 0 | 0 | 12 | 0 | 0 | 0 |
| S2_Rubber | 0 | 1 | 73 | 6 | 1 | 0 | 0 | 31 | 0 | 38 | 111 | 0 | 0 | 0 |
| S3_Rainforest | 0 | 0 | 27 | 0 | 0 | 0 | 0 | 38 | 0 | 3 | 59 | 23 | 0 | 9 |
| S3_Rainforest | 0 | 0 | 37 | 0 | 3 | 1 | 0 | 0 | 0 | 6 | 92 | 2 | 0 | 3 |
| S3_Rainforest | 0 | 0 | 19 | 0 | 4 | 11 | 0 | 0 | 0 | 10 | 141 | 20 | 1 | 0 |
| S3_Oil palm | 2 | 17 | 0 | 10 | 3 | 0 | 0 | 0 | 1 | 13 | 51 | 12 | 1 | 0 |
| S3_Oil palm | 0 | 0 | 0 | 4 | 0 | 3 | 0 | 0 | 2 | 0 | 31 | 0 | 0 | 0 |
| S3_Oil palm | 1 | 0 | 0 | 3 | 1 | 0 | 0 | 0 | 3 | 0 | 0 | 0 | 0 | 0 |
| S3_Oil palm | 0 | 5 | 55 | 25 | 6 | 2 | 0 | 0 | 0 | 0 | 35 | 0 | 0 | 0 |
| S3_Rubber | 1 | 0 | 81 | 1 | 0 | 0 | 0 | 0 | 0 | 22 | 29 | 0 | 0 | 0 |
| S3_Rubber | 0 | 3 | 74 | 4 | 0 | 0 | 0 | 7 | 0 | 3 | 32 | 0 | 2 | 1 |
| S3_Rubber | 0 | 2 | 0 | 14 | 6 | 0 | 0 | 0 | 0 | 1 | 18 | 0 | 0 | 0 |
| S3_Rubber | 0 | 0 | 40 | 5 | 1 | 0 | 20 | 4 | 0 | 8 | 14 | 5 | 0 | 0 |
| S4_Rainforest | 0 | 1 | 0 | 1 | 0 | 1 | 0 | 0 | 0 | 2 | 41 | 4 | 0 | 0 |
| S4_Rainforest | 0 | 0 | 3 | 0 | 0 | 1 | 0 | 0 | 0 | 1 | 17 | 0 | 0 | 0 |
| S4_Rainforest | 0 | 0 | 2 | 0 | 1 | 0 | 0 | 0 | 1 | 4 | 33 | 0 | 0 | 0 |
| S4_Rainforest | 0 | 0 | 18 | 0 | 0 | 5 | 0 | 0 | 0 | 0 | 22 | 4 | 0 | 0 |
| S4_Oil palm | 7 | 0 | 0 | 1 | 0 | 0 | 0 | 2 | 10 | 0 | 18 | 0 | 0 | 0 |
| S4_Oil palm | 0 | 0 | 0 | 1 | 0 | 0 | 0 | 0 | 0 | 0 | 21 | 2 | 0 | 0 |
| S4_Oil palm | 0 | 0 | 0 | 0 | 0 | 0 | 0 | 0 | 3 | 0 | 0 | 0 | 0 | 0 |
| S4_Oil palm | 0 | 0 | 0 | 1 | 0 | 0 | 0 | 3 | 0 | 3 | 32 | 0 | 0 | 1 |
| S4_Rubber | 0 | 0 | 1 | 0 | 0 | 0 | 0 | 9 | 0 | 25 | 6 | 0 | 0 | 0 |
| S4_Rubber | 0 | 0 | 16 | 1 | 0 | 0 | 0 | 6 | 0 | 16 | 3 | 0 | 0 | 1 |
| S4_Rubber | 0 | 0 | 0 | 1 | 0 | 0 | 0 | 0 | 0 | 0 | 9 | 0 | 0 | 0 |

*Acr=Acrocyrtus sp.1, All=Alloscopus tetracanthus, Asc=Ascocyrtus cinctus, Folc=Folsomides centralis, Folsp=Folsomides parvulus, Foln=Folsomina onychiurina, Hom=Homidia cingula, Isolu=Isotomiella cf. alulu, Isodes=Isotomodes sp.1, Mega=Megalothorax cf.minimus, Pseu1=Pseudosinella sp.1, Ram=Rambutsinella sp.1, Sphy= Sphyroteca sp.1, Xen1= Xenylla sp.1*

**Appendix S5.** Model selection for Collembola abundance as affected by land-use system, layer and season with plot included as random factor.

model: abundance ~ 1 + Layer * Season * System + (1 | plotID)

|  | npar | AIC | BIC | LogLik | deviance | Chisq | Df | Pr(>Chisq) |
| --- | --- | --- | --- | --- | --- | --- | --- | --- |
| model | 26 | 5643 | 5765.5 | -2795.5 | 5591 | 18.89 | 6 | 0.0044 |

Response: abundance

|  | Chisq | Df | Pr(>Chisq) |
| --- | --- | --- | --- |
| Layer | 0.06 | 1 | 0.7968 |
| Season | 9.50 | 3 | 0.0233 |
| System | 48.20 | 2 | 0.0001 |
| Layer × Season | 11.58 | 3 | 0.0089 |
| Layer × System | 16.11 | 2 | 0.0003 |
| Season × System | 19.97 | 6 | 0.0028 |
| Layer × Season × System | 19.61 | 6 | 0.0032 |

**Appendix S6.** Wilks’ lambda and p-value of LDA from every sampling date and layer

| Sampling date | Layer | Wilks’ lambda | approx. F |
| --- | --- | --- | --- |
| March | Litter | 0.045 | 14.74***** |
| June | Litter | 0.001 | 127.10***** |
| August | Litter | 0.09 | 8.78***** |
| November | Litter | 0.07 | 11.19***** |
| March | Soil | 0.24 | 4.18**** |
| June | Soil | 0.02 | 31.38***** |
| August | Soil | 0.03 | 17.47***** |
| November | Soil | 0.09 | 9.61***** |

*Chi-square values; * p < 0.05; ** p < 0.01; *** p < 0.001*

**Appendix Figure 1a**. Jambi airport monthly temperature and mean accumulated precipitation in 1991-2020 (30-years average)

**Appendix Figure 1b**. Jambi airport monthly temperature and accumulated precipitation in 2017
